# Intestinal Fibrogenesis in Inflammatory Bowel Diseases: Exploring the Potential Role of Gut Microbiota Metabolites as Modulators

**DOI:** 10.3390/ph17040490

**Published:** 2024-04-11

**Authors:** Sara Cicchinelli, Stefania Gemma, Giulia Pignataro, Andrea Piccioni, Veronica Ojetti, Antonio Gasbarrini, Francesco Franceschi, Marcello Candelli

**Affiliations:** 1 Department of Emergency, S.S. Filippo e Nicola Hospital, 67051 Avezzano, Italy; cicchinelli.sara90@gmail.com; 2 Department of Emergency, Anesthesiological and Reanimation Sciences, Fondazione Policlinico Universitario A. Gemelli, IRCCS, 00168 Rome, Italy; 3 Department of Translational Medicine and Surgery, Fondazione Policlinico Universitario A. Gemelli, IRCCS, 00168 Rome, Italy

**Keywords:** gut microbiota, gut metabolites, postbiotics, intestinal inflammation, intestinal fibrosis, epithelial-to-mesenchymal transition, EMT, inflammatory bowel disease, IBD, Crohn’s disease, ulcerative colitis

## Abstract

Fibrosis, sustained by the transformation of intestinal epithelial cells into fibroblasts (epithelial-to-mesenchymal transition, EMT), has been extensively studied in recent decades, with the molecular basis well-documented in various diseases, including inflammatory bowel diseases (IBDs). However, the factors influencing these pathways remain unclear. In recent years, the role of the gut microbiota in health and disease has garnered significant attention. Evidence suggests that an imbalanced or dysregulated microbiota, along with environmental and genetic factors, may contribute to the development of IBDs. Notably, microbes produce various metabolites that interact with host receptors and associated signaling pathways, influencing physiological and pathological changes. This review aims to present recent evidence highlighting the emerging role of the most studied metabolites as potential modulators of molecular pathways implicated in intestinal fibrosis and EMT in IBDs. These studies provide a deeper understanding of intestinal inflammation and fibrosis, elucidating the molecular basis of the microbiota role in IBDs, paving the way for future treatments.

## 1. Overview of Inflammatory Bowel Diseases (IBDs)

Inflammatory bowel diseases (IBDs), encompassing Crohn’s disease (CD) and ulcerative colitis (UC), represent a significant global health challenge due to their chronic and relapsing nature, affecting millions of individuals worldwide. The pathogenesis of IBDs is recognized as immunomediated and is still being investigated. These disorders occur in genetically predisposed individuals exposed to certain environmental and lifestyle factors, leading to dysregulated activation of the intestinal immune system (IIS) [1]. These conditions impose a substantial burden on healthcare systems, patients, and caregivers, leading to impaired quality of life and increased morbidity and mortality rates.

### 1.1. Epidemiology and Global Prevalence

IBDs have witnessed a steady rise in prevalence worldwide reaching 0.3–0.5% of the global population, with Western countries experiencing particularly high rates of incidence. However, emerging data suggest an increasing prevalence in developing regions, indicating a shift in the global burden of these diseases. Epidemiological studies highlight the significant economic and social implications of IBDs, with substantial healthcare costs attributed to disease management, hospitalizations, and a loss of productivity [2,3].

### 1.2. Clinical Manifestations and Disease Course

CD and UC exhibit different clinical manifestations and disease courses, although they share common features such as abdominal pain, diarrhea, rectal bleeding, and weight loss. CD can affect any part of the gastrointestinal tract, leading to transmural inflammation, whereas UC primarily involves the colon and rectum, causing mucosal inflammation and ulceration. The chronic and relapsing nature of IBDs contributes to long-term complications, including strictures, fistulas, colorectal cancer, and extra-intestinal manifestations, further exacerbating the disease burden [4,5].

## 2. Purpose of this Review: Are the Microbiota and Its Metabolites Key Players in Fibrogenesis in IBDs?

It is well established that the intestinal microbiota has a central role in the pathogenesis of IBDs by influencing all components of the intestinal barrier. While acknowledging inflammatory pathways as key contributors to tissue damage, recent years have witnessed a significant shift toward investigating the microbiota as a primary trigger. This shift in research focus has also revealed that many bacterial end-products may play a crucial role in modulating intestinal inflammatory pathways. Given the close interconnection between inflammatory and fibrotic pathways, it is reasonable to speculate that certain metabolites may influence the development of fibrosis in IBDs.

This review aims to explore the role of microbial metabolites in modulating the intricate molecular pathways involved in the development of epithelial-to-mesenchymal transition (EMT) and fibrosis in IBDs. Additionally, it seeks to investigate how recent discoveries regarding postbiotics may impact the therapeutic landscape for CD and UC.

## 3. The Intestinal Barrier in Health and in IBDs

The intestinal barrier serves as a critical interface between the internal milieu of the body and the external environment of the gut lumen. Comprising various components such as the microbiota, mucus layer, epithelial cells, and mucosal immune system, it selectively absorbs nutrients while preventing the entry of harmful pathogens and antigens (immune tolerance) [6]. In IBDs, the integrity and functionality of the intestinal barrier are compromised.

### 3.1. Dysbiosis: Alterations in the Intestinal Microbiota

The human microbiota consists of 10–100 trillions of microorganisms, predominantly found in the gut, with over 1000 bacterial species contributing to a microbiome of 3 million genes. Maintaining an optimal host–microbiota interaction, termed eubiosis, is crucial for normal metabolic and immune functions, preventing disease development [7]. In fact, the normal intestinal microbiota and its derivatives, including its fragments and metabolites, represent a fundamental element of the intestinal barrier. Not only do they act as a dynamic line of defense against external aggressions, preventing the proliferation and adhesion of pathogens to the intestinal mucosa, but they also actively contribute to regulating the functionality of the intestinal mucus and epithelium, maintaining the structural integrity of the barrier, and promoting proper nutrient absorption. At the same time, the microbiota plays a critical role in modulating the local immune response, contributing to the promotion of immune tolerance and the prevention of undesired inflammatory and autoimmune reactions.

The dominant gut microbial phyla are *Firmicutes*, *Bacteroidetes*, *Actinobacteria*, *Proteobacteria*, *Fusobacteria*, and *Verrucomicrobia*, with *Firmicutes* and *Bacteroidetes* constituting 90% of the gut microbiota. Dysbiosis, characterized by shifts in microbial composition and diversity, is a hallmark feature of IBDs [8]. Studies have demonstrated alterations in the relative abundance of bacterial phyla, with a decrease in beneficial commensal bacteria, such as *Firmicutes*, and an expansion of potentially pathogenic species, including *Proteobacteria* (Table 1). Dysbiosis is associated with functional changes in microbial metabolism, aberrant immune activation, and disruption of host–microbiota interactions, further exacerbating intestinal inflammation in IBDs.

### 3.2. Impairment of the Mucus Layer

The mucus layer is composed of mucins and anti-microbial peptides (AMPs). Mucins, produced by goblet cells, consist of O-linked glycoproteins and include secreted MUC2 forming polymeric networks, and transmembrane molecules like MUC1, MUC3, MUC4, and MUC13 forming the glycocalyx [26]. The mucus layer protects the intestinal epithelium from luminal antigens and maintains epithelial barrier function. In IBDs, alterations in mucin production, glycosylation, and distribution lead to impaired mucus barrier integrity, facilitating bacterial adhesion, epithelial contact, and immune activation. Bacterial species like *Akkermansia*, *Lactobacillus*, and *Bifidobacterium* spp., impair mucus secretion, composition, and thickness. Cytokines like TNF-α, IL-β, IL-4, and IL-10 modulate mucin secretion. Differences in mucus disruption can be observed between the two IBDs: in UC, the mucus layer is thinner due to reduced goblet cell function, while in CD, the mucus layer is normal or thicker with increased MUC2 expression and goblet cell hyperplasia [4,26].

### 3.3. Epithelial Dysfunction and Increased Permeability: The “Leaky Gut”

The intestinal epithelium forms a physical barrier that regulates the selective transport of nutrients, ions, and water while preventing the translocation of luminal antigens and pathogens. In particular, the intestinal epithelial cells (IECs) create a semi-permeable barrier maintained by the apical junctional complex (APC), consisting of adherens junction proteins (e.g., E-cadherin), tight junction proteins (i.e., zonula occludens, claudins, and occludin), and desmosomes. In IBDs, pro-inflammatory cytokines like TNF-α and IFN-γ promote the internalization of junctional proteins, promoting impaired cell–cell adhesion and increased epithelial permeability, resulting in a “leaky gut” [27]. Increased paracellular flux of luminal antigens, microbial products, and inflammatory mediators across the epithelium exacerbates mucosal inflammation and perpetuates the disease process in IBDs. In fact, it is useful to remark that IECs are also crucial intermediaries between the “microbiological barrier”—i.e., microbiota and its derivatives—and the immune barrier. They express pattern-recognition receptors (PRRs), including Toll-Like Receptors (TLRs), responding to microbial elements (microbe-associated molecular patterns, MAMPs) and other immune-related receptors like the aryl hydrocarbon receptor (AhR). Moreover, in addition to their primary functions, enterocytes, goblet cells, and M cells act as antigen-presenting cells (APCs) [28].

### 3.4. Dysregulation of Mucosal Immunity

**Figure 1:**
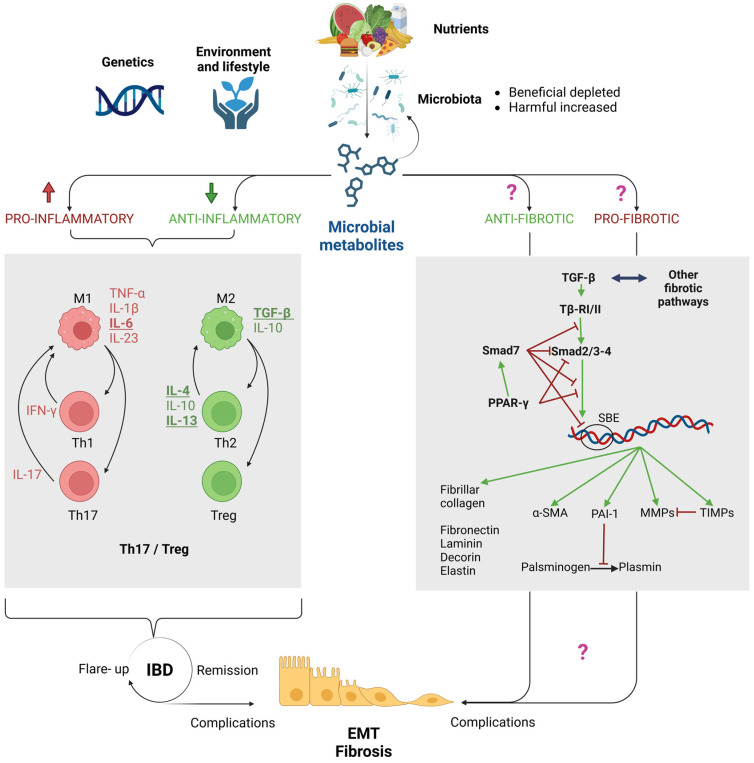
Modulation of EMT and fibrosis in inflammatory bowel diseases (IBDs) by microbial metabolites. The effect of microbial metabolites encompasses both direct and indirect pathways. Dysbiosis alters the metabolism of ingested nutrients, impacting the levels of metabolites, with diverse effects on the well-known inflammatory mechanisms underlying the pathogenesis of IBDs (left gray box). While inflammation directly influences the clinical manifestations of IBDs, it also indirectly regulates EMT and fibrosis pathways through the induction of many pro- and anti- fibrotic cytokines (underlined molecules). Although evidence supports the direct anti-fibrotic and pro-fibrotic effects of some metabolites in various organs, their specific role in IBD-related gut complications remains less understood (purple question marks), and initial evidence has been reported for a limited number of metabolites, mainly impacting on TGF-β/Smad pathway and interconnected fibrotic pathways (right gray box). Legend: green arrow: stimulates; red line: inhibits. Abbreviations: α-SMA, alpha smooth muscle actin; MMPs, matrix metalloproteinases; PAI-1, plasminogen activator inhibitor-1; PPAR-γ, peroxisome proliferator-activated receptor gamma; SBE, Smad Binding Elements; TGF-β, transforming growth factor beta; Tβ-RI/II, TGF-β receptor I/II; TIMPs, tissue inhibitors of metalloproteinases. Figure created with BioRender.com.

Mucosal immunity, orchestrated by a sophisticated interplay of innate and adaptive immune cells, maintains gut immune homeostasis and regulates responses to luminal antigens. In IBDs, under the multifactorial influx of genetic predisposition, environmental and lifestyle factors, and impaired host–microbiota interaction, dysregulation of mucosal immune responses leads to aberrant activation of innate immune cells, predominantly macrophages and T lymphocytes, resulting in excessive and harmful inflammation [29,30,31]. This dysregulation contributes to tissue damage, fibrosis, and disease progression in IBDs (Figure 1), with variations observed between CD and UC based on disease phase, elucidating clinical differences between the two conditions.

In UC, the acute phase is typified by elevated levels of pro-inflammatory cytokines (such as TNF-α, IL-1β, and IL-6), driving a Th1-type response, with M1-polarized macrophages amplifying inflammation. During the chronic phase, there is a shift toward Th2-type cytokines (IL-4 and IL-13), recruiting M2-type macrophages that intensify the Th2 response. Prolonged Th2 activation, influenced by mediators like IL-13, contributes to chronicity and pro-fibrotic mechanisms, sustained by other cytokines like IL-4 and IL-33 [32,33,34,35]. In CD, the predominant mechanism appears to involve innate immune response deficits, characterized by reduced secretion of TNF-α, IFN-γ, and IL-6 by macrophages, possibly due to dysfunctional secretory proteins linked to genetic polymorphisms [36]. This leads to CD4+ lymphocyte responses, particularly Th1 polarization, contributing to a profoundly pro-inflammatory microenvironment characterized by increased IL-12 and IFN-γ levels [37].

Overall, in both IBDs, an altered Th17/Treg balance is evident (Figure 1). Th17 cells, crucial for defense against bacteria and fungi, as well as mucosal repair and homeostasis, play a pivotal role in IBD pathogenesis, primarily influenced by pro-inflammatory cytokines like IL-6 and IL-23 [38]. These cytokines, released by macrophages, confer resistance to apoptosis in Th17 lymphocytes and induce Th1 and Th17 responses via the JAK-STAT pathway. Elevated IL-17 and IL-22 levels, produced by Th17 cells, initially promote epithelial integrity but may induce pro-inflammatory patterns and tissue damage in prolonged inflammation, potentially leading to chronic inflammation and neoplastic tissue degeneration in CD and UC patients [39]. At the same time, while the immune system mounts an inflammatory response to combat perceived threats, the anti-inflammatory mechanisms aimed at resolving inflammation are often ineffective in IBDs. Regulatory T cells (Tregs), through the secretion of cytokines like IL-10 and TGF-β, play a crucial role in immune tolerance and suppression of excessive inflammation. These cells may become dysfunctional or insufficient in IBD patients [40].

## 4. The Role of Immune Dysregulation in Driving Epithelial-to-Mesenchymal Transition (EMT) and Fibrosis in IBDs

As discussed above, in IBDs, the equilibrium between pro-inflammatory and anti-inflammatory cytokines is disrupted, leading to chronic inflammation. This chronic pro-inflammatory stimulus is counteracted by anti-inflammatory mechanisms that may temporarily quell inflammation and alleviate symptoms. Intriguingly, the same mechanisms intended to facilitate inflammation resolution and mucosal healing become aberrant, contributing to tissue damage (Figure 1). In this context, the altered Th17/Treg ratio affects the concentration of the pleiotropic cytokines IL-10 and TGF-β in the different disease phases. TGF-β may be reduced during acute phases due to the presence of pro-inflammatory cytokines. This reduction may exacerbate inflammation and contribute to disease flares. However, during phases of remission or tissue healing, elevated levels of TGF-β have been observed, especially during fibrosis and tissue repair stages [41].

TGF-β belongs to a vast superfamily [42], whose members bind to transmembrane receptors of two types, type I (TβR-I) and type II (TβR-II), containing a serine/threonine kinase cytoplasmic domain. The ligand-receptor binding activates Smad-dependent signaling, called the “canonical pathway”, well reviewed elsewhere [43,44] (Figure 1). Synthetically, the binding of a ligand with two TβR-I and two TβR-II receptors induces the formation of a heterotetrameric complex, which finally phosphorylates adaptor proteins called regulatory Smads (R-Smads), Smad2/3 pathway for TGF-βs, or Smad1/5/8 for other family members like Bone Morphogenetic Proteins (BMPs). The subsequent interaction with a co-adaptor (Smad4) allows for their translocation in the nucleus, their interaction with target genes, and their transcription. The translocation is inhibited by the inhibitory Smads (I-Smads), namely, Smad6 and Smad7 [44]. Besides the “canonical pathway”, some TGF-β superfamily members may also modulate Smad-independent “non-canonical pathways”, adding a layer of complexity to the regulation of cellular responses to TGF-β [45]. Among them we include the PI3K (PI3K, phosphatidylinositol-3-kinase), and MAPK (mitogen-activated protein kinase) pathways. Beside TGF-β, these pathways are activated by many growth factors, like Epithelial Growth Factor (EGF), Fibroblast Growth Factor (FGF), and Platelet-Derived Growth Factor (PDGF) for the MAPK pathway, and IGF-1 (Insulin-like Growth Factor-1) for the PI3K pathway, respectively [46,47].

As TGF-β is a pleiotropic cytokine, its transduction pathways influence the transcription of three main categories of genes: (1) genes involved in embryogenesis, cell proliferation, and differentiation; (2) immunomodulatory genes; (3) genes encoding proteins, enzymes, and growth factors involved in extracellular matrix (ECM) homeostasis, and subsequently acting on the mechanisms of healing and tissue repair.

While its role as an immunomodulatory agent has already be outlined in the previous paragraphs, TGF-β also maintains the barrier integrity by a regulated expression of epithelial tight-junction proteins and by the transcription of ECM structural proteins, like fibrillar collagen, as well as those of fibronectin, laminin, decorin, elastin, α-SMA (alpha smooth muscle actin), and many others [48]. Together with other cytokines, it also modulates the equilibrium of the breakdown/deposition of matrix proteins, through the balance between matrix-degrading proteases and their inhibitors such as PAI-1 (Plasminogen Activator Inhibitor-1), TIMP1 and TIMP2 (Tissue Inhibitors of Metalloproteinases), and MMPs (metalloproteinases) [49,50] (Figure 1).

The altered secretion and function of TGF-β and its interconnected pathways are one of the most studied triggers for the so called epithelial-to-mesenchymal transition (EMT) and fibrosis development. Thus, in certain conditions, it could also be considered a pro-fibrotic cytokine.

EMT is a biological process in which epithelial cells lose their characteristic features and acquire mesenchymal cell properties. Mesenchymal cells include fibroblasts, myofibroblasts, and smooth muscle cells [51]. Normally, EMT is a process in which the transient appearance of mesenchymal cells contributes to normal wound healing. In addition to this role, EMT is involved in embryogenesis and oncogenesis [52], and various pathological settings characterized by fibrosis. In fact, EMT becomes harmful when the dysregulation of the repair mechanisms, triggered by chronic inflammation and sustained by factors like TGF-β, EGF, FGF, PDGF, and IGF-1, results in the persistent loss of typical epithelial features. These changes consist of a loss of cell adhesion and polarity, while gaining the ability to migrate and invade, as well as the capacity to deposit excessive ECMs. These events have been described in various organs, including the cardiovascular system [53], kidneys [54], and lungs [55], and the intestine [56]. Additionally, it is noteworthy that besides EMT, mesenchymal-to-epithelial transition (MET) is possible and contributes to wound repair and oncogenesis [57].

The above explained dual role of TGF-β, as an immunomodulatory and pro-fibrotic cytokine, is pivotal in the pathogenesis of IBDs. On the one hand, although overexpressed, it loses its anti-inflammatory ability because of a reduction in the number of Treg cells and their inadequate action, together with the overexpression of the inhibitory Smad7 [56]. On the other hand, the prolonged action of TGF-β during the chronic evolution of the disease correlates with the fibrotic complications [58].

Interestingly, TGF-β/Smad effects are counteracted—beside the effects of I-Smads [44]—by the peroxisome proliferator-activated receptor gamma (PPAR-γ). PPAR-γ is a member of the ligand-activated transcription factors of the nuclear hormone receptor superfamily, with pleiotropic effects on lipid metabolism, inflammation, cell proliferation, and fibrosis [59]. PPAR-γ expresses its anti-inflammatory and anti-fibrotic effects on the TGF-β pathway in many ways. It can enhance the inhibitory effect of Smad7 or inhibit Smad2/3 interaction with Smad4. Moreover, it blocks the nuclear translocation of the Smad2/3/4 complex [60] (Figure 1). Significantly impaired PPAR-γ expression is observed in colonic epithelial cells of IBD patients, suggesting that the disruption of PPAR-γ signaling may represent a critical step of the IBD pathogenesis [61]. Overexpression of PPAR-γ prevents tissue fibrosis, whereas its loss increases fibrosis [59].

It should be noted that while the most extensively studied pathway implicated in ECM deposition and EMT is the canonical TGF-β pathway, an expanding area of investigation in the pathogenesis of IBDs relates to alternative pathways including other TGF-β superfamily members (especially BMPs), the non-canonical pathways, TLR receptors (TLRs), and the pregnane X receptor (PXR). BMPs can impact the differentiation of stromal cells contributing to fibrosis, while activins and inhibins influence the production of ECM components [45,62,63]. TLRs, the main exponents of the PPR (Pattern Recognition Receptor) family, detect pathogens and damaged cells through pathogen- and damage-associated molecular patterns (PAMPs and DAMPs). They are expressed on immune cells and their activation results in the secretion of pro-inflammatory cytokines (including IL-1, IL-6, IL-8, TNF, IL-33, and IFN-γ) [64,65] and in the regulation of the balance between Th1 and Th2 immune responses [66]. Beside their pro-inflammatory role, some studies have suggested that TLR2 and TLR4 are also involved in EMT through the modulation of the TGF-β pathway [67]. The PXR, although less studied, is still known for its involvement in skin and liver fibrosis and has been suggested as a novel mediator of intestinal fibrosis, with relevant involvement of the mesenchymal compartment. As reported in animal models of colitis, its loss results in protracted inflammation and fibrosis [68].

## 5. Microbial Metabolites: Established Players in Intestinal Homeostasis and IBD Inflammation, with Potential Implications in Fibrosis

Intestinal microbial metabolites can potentially be derived from all macronutrients present in food through complex metabolic mechanisms, including fermentation, decarboxylation, hydrolysis, and others. It is important to introduce the concept of “postbiotics” which, as reported in the consensus statement of the International Scientific Association of Probiotics and Prebiotics (ISAPP), are a “preparation of inanimate microorganisms and/or their components that confers a health benefit on the host” [69]. This definition remarks how the benefit of the host is a mandatory characteristic for postbiotics, which is also shared by pro- and postbiotics, and outlines how not all of the microbial metabolites can be considered postbiotics, since some of them can be harmful to the host.

In the last few years, the role of microbial metabolites in maintaining the homeostasis of the healthy gut barrier has been elucidated (Table 2).

Table 1 outlines the alterations in microbiota composition observed in IBDs, while Table 2 delineates the primary species responsible for synthesizing specific metabolites. Integrating this information reveals how changes in microbial composition disrupt the equilibrium between detrimental and beneficial metabolites, consequently impacting various components of the intestinal barrier. While the effects of microbiota-derived metabolites on modulating inflammatory pathways have been extensively studied and have even led to some therapeutic applications, the existence of self-perpetuating, microbiota- and metabolite-related fibrosis mechanisms remains less clear and constitutes an area of research to explore, particularly given the varying responses to anti-inflammatory therapies among IBD patients and the lack of anti-fibrotic therapies.

### 5.1. Short-Chain Fatty Acids (SCFAs)

SCFAs are essential for maintaining gut homeostasis. As indicated in Table 1 and Table 2, certain bacterial species such as *Clostridiales* (e.g., *F. prausnitzii*) and *Bacteroidetes*, which are involved in SCFA metabolism, are depleted in IBDs, leading to reduced SCFA levels in both CD [95] and UC [96]. This depletion highlights the significance of SCFAs in IBD pathogenesis, culminating in the fact that interventions such as the administration of butyrate-related pre- and probiotics or topical/oral butyrate supplementation have been found to be beneficial in managing these diseases, both in preclinical and clinical models [97,98,99,100]. The beneficial effects are largely attributed to the anti-inflammatory properties of SCFAs, particularly butyrate. For instance, butyrate has been shown to modulate Tregs, reduce the production of pro-inflammatory cytokines such as TNF-α, IL-1β, and IL-6, promote the differentiation of M2 macrophages, and inhibit M1 macrophages and neutrophils [101,102,103,104].

Regarding the involvement of SCFAs in fibrosis pathways, most evidence comes from extra-intestinal studies. A study on renal cells exposed to elevated concentrations of butyrate reported the suppression of TGF-β1 synthesis and signaling [105]. The administration of a butyrate-producing strain not only attenuated cisplatin-induced renal inflammation but also decreased the expression of ECM molecules like collagen IV, fibronectin, and α-SMA [106]. Similarly, in preclinical models of diabetic nephropathy, the administration of sodium butyrate reduced TGF-β1-induced fibrosis, with lower deposition of ECM proteins like collagen, fibronectin, and α-SMA [107]. In the heart, butyric acid ameliorated fibrosis by regulating M1/M2 polarization of macrophages [108], or by targeting the deposition of collagen [109]. In the liver, different SCFAs exert different effects, and regarding fibrosis, sometimes contrasting results have been found. For example, high doses of propionate are known to be hepatotoxic and are used to induce liver fibrosis in animal models [110]. In some studies, butyrate and acetate have shown anti-fibrotic properties through the deactivation of TGF-β signaling and of some non-canonical TGF-β pathways [111,112]. Other authors have found that in patients with metabolic dysfunction-associated steatotic liver disease (MASLD), higher serum levels of propionate (*p* = 0.02) and butyrate (*p* = 0.03) were associated with fibrosis severity. In such patients, gut dysbiosis has been reported—with *Ruminococcaceae* and *Veillonellaceae* as the main microbial taxa associated with significant fibrosis—and stool propionate levels are significantly elevated, in correlation with fibrosis severity [113]. These discrepancies might be explained by the fact that SCFAs are normally absorbed in the liver, and higher serum levels potentially reflect the impaired hepatic function in cirrhosis or the existence of portosystemic shunts [114]. In the lungs, propionate was found to reduce EMT in alveolar epithelial cells through the inhibition of PI3K/Akt/mTOR signaling [115].

An indirect role of butyrate and its related microbiota in fibrosis pathways has been attributed to the modulation of TLRs and macrophage polarization [116]. Among other possible indirect anti-fibrotic effects of butyrate, we cite its ability to inactivate the histone-deacetylases (HDACs) and activate the histone-acetylases (HATs), or to influence the histone butyrylation, acting as a regulator of the epigenetic processes, possibly influencing the fibrotic mechanisms too [117,118]. Moreover, it is an emerging AhR ligand, a receptor that has been proposed as a pivotal mediator of diet–microbiota–host interaction thanks to its duplex ability to recognize many xenobiotic compounds and modulate immune cell function [119]. Furthermore, other receptors and pathways may contribute to the anti-fibrotic effects of SCFAs. For instance, G-protein-coupled receptors (GPRs) and free fatty acid receptors (FFARs) have been documented to modulate the TGF-β pathway and ECM deposition in different organs [120,121,122].

Unfortunately, the direct role of SCFAs in EMT and fibrosis in the gut remains elusive and little, inconclusive evidence is available. In the intestine, SCFAs producing *Clostridiales* promote a TGF-β1-rich environment by stimulating its secretion by Tregs and by IECs [123]. In the colon, the ability of butyrate to induce PPAR-γ has been described, and linked to a subsequent reduction in *Enterobacteriaceae*, especially *E. coli* [124,125,126]. Interestingly, some bacterial species which have been found to inhibit colitis and strongly activate the butyrate-related PPAR-γ induction—namely, *R. hominis* and *R. intestinalis*—are depleted in the gut of patients with IBDs [127,128]. Additionally, a protective role against colorectal cancer has been suggested, depending on the ability of butyrate to induce Smad3, enhancing TGF-β-mediated repression of the inhibitors of differentiation (Ids), with subsequent apoptosis [129]. It is worth noting that the role of butyrate and butyrate-producing bacteria, such as *C. butyricum* and *F. prausnitzii*, has also been examined concerning their contribution to EMT in oncogenesis. Indeed, studies have reported that butyrate could potentially mitigate the development of colorectal cancer, a formidable complication of UC, by modulating the Wnt/β-catenin pathway [130].

### 5.2. Lactic Acid (LA)

Lactic acid bacteria (LAB) appear to have an anti-inflammatory function (Table 2) that has been demonstrated in both CD and UC [131]. The effect seems greater for UC, in which LAB promote the induction and maintenance of remission by promoting the shift of macrophages from M1 to M2, reducing the pro-inflammatory TNF-α and NF-κB signaling and increasing the anti-inflammatory IL-10, inhibiting the inflammasome, and modulating the gut microbiota by favoring the selection of beneficial species, resulting in increased SCFA content [132,133].

The role of LA in fibrotic pathways has been explored in various organs. For example, Kottman et al. demonstrated how LA can promote myofibroblast differentiation in patients with idiopathic pulmonary fibrosis by activating TGF-β [134]. Another study showed that treatment with certain LAB species would increase TGF-β expression in asthmatic patients and be able to reduce eosinophilic airway infiltration [135]. In a rat model of thioacetamide-induced liver fibrosis, the oral administration of a mixture of LAB (*L. paracasei*, *L. casei*, and *W. confuse*) produced a protective effect, characterized by a significant reduction in the deposition of collagen and α-SMA, and a decreased concentration of TGF-β [136]. In the skin, some LAB seem to be photoprotective thanks to their ability to modulate MMPs in fibroblasts [137,138].

At the intestinal level, the oral administration of *L. gasseri* appears to have beneficial effects on the mucosa as it stimulates the release of IgA from DCs through the activation of TGF-β [139]. The administration of fermented soymilk to a preclinical model of DSS-induced colitis showed that LAB enhanced the growth of SCFA-producing bacteria, with an increase in PPAR-γ and a subsequent reduction in the inflammatory cytokines [140]. Although there are few studies analyzing the relationship between LA and pro-fibrotic patterns at the intestinal level, we speculate that LA may protect the gut from acute inflammatory responses in part by up-regulating anti-inflammatory pathways such as TGF-β, and in part by decreasing the fibrosis mediated by this cytokine. In the context of IBDs, this speculation is in line with the evidence that species belonging to LAB groups are decreased in those patients, especially in the ones affected by UC (see Table 1). However, it should be noted that while human cells mainly produce L-lactate, LAB can produce D-lactate, levels of which have been correlated with disease activity and serum inflammatory markers in IBDs [141]. This evidence suggests that the role of lactic acid and LAB in inflammation and fibrosis might also depend on the balance between different isoforms of lactate.

### 5.3. Indoles

Indoles are produced by intestinal microbiota from the essential amino acid tryptophan (Trp). In the human body, the microbiota-mediated indole pathway is interconnected with other two endogenous Trp metabolic pathways, the kynurenine and the serotonin pathways. Indoles have been found to normally modulate the function of the gut barrier (Table 2) and to exert both beneficial and detrimental effects in various organs through the modulation of inflammatory and fibrotic pathways (Table 3).

In the context of active IBDs, reduced Trp absorption, heightened activity in the kynurenine pathway, elevated availability of interstitial serotonin, modifications to the indole pathway, and activation of signaling through the AhR have been revealed. Once again, as reported for SCFAs and LA, most of the beneficial effects of indoles in mitigating the IBDs reside in the protective effects on the components of intestinal barrier, having anti-inflammatory effects [142,143]. For example, it has been observed that dietary Trp deficiency promotes DSS-induced inflammation, while it is alleviated by Trp supplementation [144]. Trp availability could decrease because of the depletion of some bacterial species (Table 1), with subsequent reduction in beneficial indoles that have shown the ability to affect non-Trp-producing bacteria’s (e.g., *C. albicans*, *S. enterica*, *P. aeruginosa*, *E. coli*, and *Klebsiella* spp.) invasiveness, motility, and toxicity. Moreover, these alterations might affect mucus composition and the intercellular tight junctions, contributing to the mechanism of a leaky gut [78].

Regarding the direct anti-fibrotic effects, in a distinguished study by Flannigan et al., conducted in a mice model of colitis, the supplementation of the Trp metabolite Indole-3-propionic acid (IPA) notably reduced the development of fibrosis through the modulation of the PXR receptor. In the same study, this receptor was found to be reduced in IBD patients, and their IPA fecal levels were lower when compared to healthy subjects. The authors also found a reduced expression of pro-inflammatory cytokines in human myofibroblasts challenged with LPS after a pretreatment with IPA [68]. Interestingly, some studies have revealed that diet-derived indoles, like the indole-3-carbinol (I3C) contained in some *Brassica* family vegetables and yet to be investigated for its anti-inflammatory effects through the modulation of AhR [145], can impact the synthesis of PPAR-γ in mice fed with different diets [146].

An analysis of the action of the various indoles in various organs (Table 4) suggests that since they can coexist in the same milieu, their influence on the pathways of fibrosis may depend on various factors, such as the type of diet (e.g., high-fat diet), the dietary availability of Trp-related molecules, the microenvironment and the activity of the kynurenine and serotonin pathways, the target cells, the specific modifications of the microbiota, and their final balance between pro-fibrotic and anti-fibrotic effects.

**Table 3:** Main interactions between indoles and main molecular pathways involved in inflammation and fibrosis.

	IAA	IPA	ILA	IS	IC
**AhR**		Kidney: IPA suppresses the IS effect on the receptor [147].	Gut: Supplementation with *L. acidophilus*, or its metabolite ILA, attenuates inflammation and restores IL-22 levels through AhR signaling in mice [142]. Similar results were observed in a mice model of DSS-induced colitis supplemented with two strains of ILA-producing *B. bifidum* [143].	Liver: IS is an agonist of the AhR receptor [147].	Gut: depletion of dietary IC is fatal in AhR IEC-deficient mice and worsens chronic colitis in C57BL/6 mice; in contrast, its administration reduces the Th17/Treg ratio in the same model [145,148].
**TGF-β**	Peritoneum: the novel IAA analogue MA-35 reduces TGF-β-positive cells in a murine model of peritoneal fibrosis [149].	Kidney: IPA suppresses the IS effect on the receptor [147]. Liver: IPA aggravates CCl_4_-induced fibrosis by activating TGF-β1/Smads signaling in HSCs [150].		Kidney: IS induces fibrosis through the stimulation of TGF-β1 [147].	
**Smads**	Kidney: the IAA novel analogue, MA-35, inhibits the phosphorylation of Smad3, thus reducing TGF-β1 signaling and related renal fibrosis [151].	Liver: IPA aggravates CCl_4_-induced fibrosis by activating TGF-β1/Smads signaling in HSCs [150].			
**PPAR-γ**					Adipocytes: the administration of I3C restores the levels of PPAR-γ, which were deregulated in mice fed with a high-fat diet [146].
**ECM**	Peritoneum: the treatment with the novel IAA analogue MA-35 reduces α-SMA-positive myofibroblasts in a murine model of peritoneal fibrosis [149].	Liver: IPA reduces α-SMA and collagen deposition and MMP expression while inducing TIMPs in TGF-β1-stimulated hepatic stellate cells [152]. Liver: IPA aggravates CCl_4_-induced fibrosis by activating TGF-β1/Smads signaling in HSCs [150].		Kidney: IS enhances α-SMA expression [147].	
**PXR**		Gut: IPA reduces PXR-induced fibrosis in a mice model of colitis; IBD patients showed lower levels of PXR and fecal IPA [68].			

Abbreviations: AhR, aryl-hydrocarbon receptor; α-SMA, α-smooth muscle actin; DSS, dextran Sulfate Sodium; ECM, extracellular matrix; HSCs, hepatic stellate cells; IAA, indole-3-acetic acid; IBD, inflammatory bowel disease; IC, indole-3-carbinol; IECs, intestinal epithelial cells; ILA, indole-3-lactic acid; IPA, indole-3-propionic acid; IS, indoxyl sulfate; MA-35, mitochonic acid-35; MMPs, metalloproteinases; PPAR-γ, peroxisome proliferation-activated receptor-γ; PXR, pregnane X receptor; TGF-β, transforming growth factor-β; TIMPs, tissue inhibitors of metalloproteinases. Please note that the table does not encompass all indoles produced by the intestinal metabolism of tryptophan, and only those indoles for which publications related to their involvement in fibrosis pathways have been found are included in the table.

**Table 4:** The article search conducted at the end of March 2024. The number in the first column represents the quantity of articles acquired through the PubMed query, while the bold number denotes the quantity of articles selected for review after reading the title and abstract, with duplicates removed.

QUERY		(“IBD” OR “Gut”) AND (“TGF-Beta” OR “Smad” OR “PPAR-Gamma” OR “Fibrosis” OR “EMT” OR “Alpha-SMA” OR “MMP” OR “PAI-1” OR “TIMP”)	Title and Abstract Check
	AND		
“butyrate” OR “butyric acid”		83	**16**
“acetate” OR “acetic acid”		58	**4**
“propionate” OR “propionic acid”		41	**5**
“lactic acid”		27	**1**
“indole-3-acetic acid”		5	**1**
“indole-3-carbinol”		2	**0**
“indole-3-lactic acid”		0	**0**
“indole-3-propionic acid”		5	**2**
“indoxyl sulfate”		1	**0**
“urolithin”		5	**1**
“hydrogen sulfide”		1	**0**
“trimethylamine” OR “TMAO” OR “trimethylamine-N-oxide”		52	**8**
**Total**		280	**38**

### 5.4. Urolithins (Uros)

The evidence about Uros preservation on the gut barrier and their anti-inflammatory effects is less with respect to other metabolites (Table 2). Nevertheless, the anti-fibrotic potential of UA has been observed in organs different from the gut. In a study by Chen et al. conducted on a TGF-β1-treated cardiac fibroblasts model, the administration of UA caused the activation of the Nrf2 pathway. This pathway is known for its role in redox homeostasis and anti-oxidant response, which has been proposed to counteract TGF-β-induced oxidative stress and related fibrosis [153]. The inhibitory effect of urolithin on TGF-β/Smad has been reported in the kidneys, while the induction of PPAR-γ has been described in the endothelium, but these effects have not yet been reported in the gut [154]. However, there is some evidence on the ability of ellagic acid, a precursor of urolithins, to enhance the proliferation of SCFA-producing bacteria in the gut and to activate the PPAR-γ pathway [155]. Cheng et al. reported that UA administration in lung cancer cells could affect EMT by influencing the p53-Mdm2-Snail pathway. Specifically, Snail, identified as a zinc finger transcriptional repressor, plays a role in EMT by suppressing the crucial epithelial marker, E-cadherin [156]. All of the mentioned mechanisms may contribute to mitigating inflammation [83] and fibrosis in the intestine, even if most of the available evidence comes from preclinical studies.

### 5.5. Hydrogen Sulfide (H_2_S)

There is evidence that both excessive and insufficient H_2_S levels can have various beneficial or harmful consequences on the gut barrier (Table 2). In IBDs, studies suggest that H_2_S may play a bidirectional role, with both protective and damaging effects, with a major impact in UC patients. While appropriate levels of H_2_S may have anti-inflammatory effects and contribute to maintaining intestinal mucosal integrity, elevated levels are related to inflammation and gut barrier damage, including fibrosis. For example, while the administration of the compound has been able to alleviate DSS-induced colitis by lowering the levels of pro-inflammatory IL-1β, IL-10, and TNF-α [157], other studies have suggested that the metabolic impairment that leads to lower ATP mitochondrial synthesis is a pro-inflammatory trigger in the gut of CD patients, since it leads to IL-6 production [158]. When it comes to the mechanisms of EMT and fibrosis, studies performed in several disease contexts have shown that H_2_S might affect TGF-β receptor signaling (potentially inhibitory) and PPAR-γ activity (potentially stimulatory), exhibiting varied effects on Smad proteins [159,160,161]. However, the specific impact of these pathways in the context of CD and UC is still being elucidated but it seems that the prevalent effect of H_2_S in these illnesses is the pro-inflammatory one, in line with the evidence that H_2_S-producing bacteria seem to be more abundant in both conditions (Table 1).

### 5.6. Trimethylamine (TMA) and Trimethylamine-N-Oxide (TMAO)

The role of the TMA-derived metabolite, trimethylamine-N-oxide (TMAO), has been mainly studied in cardiovascular health, since at elevated concentrations, it has been associated with a higher incidence of cardiovascular diseases, including atherosclerosis and heart disease. Interestingly, in preclinical models of cardiac failure, there is evidence that this compound may promote cardiac fibrosis through the fibroblast–myofibroblast transition mediated by the activation of TGF-β/Smad3 signaling [162,163], whilst an analogue of choline, the 3,3-dimethyl-1-butanol (DMB), has protective effects on cardiac remodeling through the inhibition of the same pathway [164]. An interesting study on 44 subjects with myocardial infarction who underwent percutaneous coronary intervention demonstrated that the administration of the probiotic *L. rhamnosus* GG strain (LGG) improved the echocardiographic indices of ventricular function and reduced the serum concentrations of cardiac remodeling biomarkers, including TMAO and TGF-β [165]. Similarly, in a preclinical model of obstructive apnea and dietary high salt intake, high levels of TMAO have been related to a depletion of gut *Lactobacilli*, a higher incidence of hypertension, and Th1 polarization of lymphocytes. The administration of LGG was able to mitigate these manifestations [166]. Profibrotic effects of TMAO have been reported also in the kidney [167,168,169], skin fibroblasts, vascular endothelial cells, adipocyte progenitor cells [170], and periodontal tissue [171]. In this context, preclinical models exposed to targeted inhibition of gut microbial TMAO production showed enhanced cardiac and renal function, also attributed to the regulation of pro-fibrotic pathways such as TGF-β [172,173]. Although there is evidence on TGF-β-related TMAO-induced fibrosis in various organs, the data on intestinal fibrosis are scarce. In the gut of IBD patients, it has been reported that there is an increased TMA/TMAO ratio, probably related to the reduction in TMA-metabolizing bacteria (Table 1) [174]. TMA exerts detrimental effects, both in vitro and in vivo, on colonic cells by causing oxidative stress-induced DNA damage, cell cycle arrest, and increased inflammatory infiltration [175]. Moreover, it activates fibroblasts toward a profibrogenic phenotype [174]. Regarding the relationship between TMAO levels and IBD predisposition and activity, the concentrations of the metabolite have been found to be reduced or normal [176,177].

## 6. Discussion: Current Knowledge and Therapeutic Perspectives of Microbiota Metabolite Modulation in Intestinal Fibrogenesis

Despite evolving data on microbiota changes in IBDs, it is widely agreed that beneficial bacteria decrease, while harmful species increase (Table 1). These changes affect various aspects of the intestinal barrier and can influence intestinal permeability and immune activation (Table 2). Although the anti- or pro-inflammatory effects of probiotics are understood, their impact on fibrogenesis remains unclear.

We included specific metabolites in this review based on evidence of their effects on inflammation and fibrogenesis: SCFAs, lactic acid, tryptophan, urolithins, hydrogen sulfide, trimethylamine, and TMAO. However, bacterial end-products encompass a wide range of compounds including LCFAs, vitamins, bile acids, endogenous alcohols, branched amino acids, flavonoids, and more, many of which are gaining attention in the context of IBDs. We distinguished two ways in which microbial metabolites modulate fibrogenesis: indirectly, through the regulation of inflammation, particularly macrophage and lymphocyte polarization [178], and directly, which is less explored in the gut but was the focus of our review (Figure 1).

Our analysis reveals limited studies with small sample sizes, often conducted in vitro or on animal models, sometimes yielding contradictory results (Table 4). Notably, while many studies investigate the role of microbial metabolites in fibrosis across distant organs, their primary production site is the intestine.

Fibrosis in various organs involves enhanced ECM deposition and EMT, mainly regulated by the canonical TGF-β/Smad pathway. However, the pleiotropic nature of the cytokine poses a “TGF-β paradox”: it can inhibit proliferation in benign cells while promoting cancer progression [179]. From our point of view, in IBDs, this paradox manifests differently as a conflict between the anti-inflammatory action of TGF-β and its pro-fibrotic effects. In fact, despite being secreted by the M2/Th2 arm of the IIS in an effort to limit the uncontrolled inflammatory burden of the M1/Th1 arm and promote the mucosal healing, the chronic inflammatory stimulus induces the pro-fibrotic changes culminating in EMT and eventually in cancer progression. This paradox implicates that TGF-β and related pathways are interesting but potentially harmful targets for new therapies.

In fact, our impression is that the existence of the TGF-β paradox has directed IBD research toward understanding inflammatory mechanisms in order to control them early on and prevent complications, including fibrosis, rather than directly influencing fibrogenesis. However, this linear view (acute inflammation—chronic inflammation—fibrosis) only partially captures the molecular complexity of IBDs, where various phenotypes can coexist.

From our literature review, despite limitations such as predominant preclinical data from studies in other organs and to a lesser extent in the intestine, several observations can be made.

The initial hypothesis that dysbiosis causes a depletion of postbiotics has been generally confirmed [95,144]. Accordingly, some studies indicate that the administration of postbiotic-producing bacteria, or of the postbiotics themselves, can reduce both inflammation and fibrogenesis in colitis models. Even if contradictory data may arise from the separate analysis of each metabolite (see Section 5), it should be considered that these alterations often coexist in patients with IBDs. Therefore, the final effect likely results from the interaction of various metabolites and related pathways, including both pro- and anti-inflammatory as well as pro- and anti-fibrotic mechanisms, which influence each other throughout the pathology stages.

Furthermore, CD and UC exhibit significant differences in immune pathways, histological and clinical manifestations, and complications, which also reflect on microbiota features. For instance, the small intestine has lower bacterial counts compared to the colon, and dysbiotic oscillations differ between CD and UC [180]. These differences may affect the production and availability of metabolites (Table 1), influencing molecular pathways and explaining various disease features. Given the higher bacterial abundance in the colon, most metabolic events occur there, with postbiotics being absorbed by the intestinal mucosa and potentially affecting distant organs via the bloodstream. For example, while SCFAs play a well-described role in UC due to their derivation from the fermentation of fibers in the colon [96], they have also been implicated in CD exacerbations [95].

The advantages of using postbiotics in preventing IBD exacerbations and complications might stem from their potentially greater stability, both during industrial processes and storage, and safety [69]. In fact, even if probiotic administration has been found to be safe, it involves the use of one or more strains of live bacteria with potentially unpredictable interactions with the recipient’s microbiota, including the extremely rare but dreaded bacteremia. Similarly, other emerging methods such as fecal transplantation entail administering material from a healthy donor, with the caveat that the composition of a “healthy” microbiota is not fully understood [7]. Instead, postbiotics supplement what is lacking in the organism without introducing living deficient microbial species. A similar rationale applies to prebiotics, which can also be supplemented, but dysbiosis might result in an unpredictable production of end-products due to a lack of species necessary to convert prebiotics into the desired molecule. Furthermore, the potential advantage lies in the fact that microbial metabolites, especially postbiotics, can trigger beneficial feedback signals on the altered microbiota, potentially promoting restoration of normal bacterial flora. An example of this principle’s applicability is the positive effect of butyrate oral or topical administration in the treatment of ulcerative colitis [97,98]. The basis for this strategy primarily lies in inflammation modulation, while from our literature review, the potential anti-fibrotic effects have not been extensively investigated.

It is noteworthy that some studies on Crohn’s disease have revealed the histological heterogeneity of intestinal strictures. While both inflammatory and fibrotic conditions can coexist, strictures may predominantly exhibit either inflammatory or fibrotic characteristics. This discrepancy suggests the need for distinct therapeutic approaches: an initial medical approach focusing on anti-inflammatory therapy might be suitable for primarily inflammatory strictures, while a surgical approach may be necessary for predominantly fibrotic strictures. Currently, there are no effective preventive or reversal medical therapies available for such strictures. Proposed strategies to avoid surgery include endoscopic ballooning or local corticosteroid injections, but these methods have high recurrence rates. However, the concept of an intermediate scenario where progression toward a fibrotic morphology of the stricture can be slowed or prevented through modulation of fibrosis pathways could generate interest in the potential anti-fibrotic effects of certain metabolites. These could serve as complementary therapy to other molecules currently under investigation, primarily monoclonal antibodies and growth inhibition factors [181]. Moreover, it has been reported that the various polymorphisms of the NOD2 gene correlate with a higher risk of surgery, suggesting that different defects in microbial sensing by NOD2 are differently predisposed to fibrosis. Despite significant therapeutic advancements targeting inflammation suppression, the occurrence of intestinal complications such as strictures and penetrations in CD patients has shown little change. These findings indicate that solely targeting inflammation may not substantially alter the clinical outcomes of intestinal fibrosis in CD patients [116]. Moreover, there is evidence that CD patients experiencing persistent symptoms in the absence of inflammation show a depletion in butyrate and indole-producing bacteria, thus suggesting that as for fibrosis, other clinical aspects of the disease might also not solely depend on inflammatory mechanisms and that microbial metabolites are once again implicated in those mechanisms [182].

In conclusion, the authors’ interest in reviewing the role of microbial metabolites in the development of fibrosis in IBDs was driven by three main considerations.

Firstly, despite the efficacy of current anti-inflammatory therapies for IBDs, a subset of patients, particularly those with CD, still experience fibrotic complications, leading to a persistent need for surgery. This discrepancy suggests that while chronic inflammation and the concomitant inadequate anti-inflammatory response are primary triggers of fibrosis and EMT, there may also be non-inflammatory pathways contributing to fibrosis that are not targeted by existing anti-inflammatory treatments.

Secondly, the absence of specific anti-fibrotic therapies for intestinal fibrosis may be attributed to the characteristics of the mechanisms underlying IBD complications, which raises concerns about the potential side effects. In fact, these mechanisms not only play a role in normal wound healing but are also implicated in processes such as embryogenesis and oncogenesis. For instance, the TGF-β pathway, also implicated in the development of colorectal cancer in UC patients, underscores the complexity of fibrotic pathogenesis in IBDs.

Thirdly, recent advances in understanding the role of the microbiota in IBDs have highlighted the impact of bacterial end-products on intestinal barrier integrity and disease manifestation. Besides the growing literature exploring the effects of these metabolites on microbiota modulation, mucus layer maintenance, epithelial functionality, and inflammatory pathways, surprisingly few studies have investigated their potential effects on fibrotic pathways. This is particularly striking considering that many of these metabolites are produced in the gut and then absorbed into the bloodstream to reach distant organs. However, research on the effects of these metabolites on EMT and fibrosis in various organs, including the lungs, heart, kidneys, and skin, has revealed a consistent trend. Metabolites known to be beneficial in the intestine, such as butyrate, lactic acid, certain indoles, and urolithin A, have generally demonstrated anti-fibrotic effects in other organs. These effects are often mediated through the modulation of regulatory factors like PPAR-γ, ultimately reducing the activity of the TGF-β/Smad pathway [105,111,124,125,136,147]. In the gut, the mechanisms remain elusive but the most consolidated evidence suggests that SCFA-producing bacteria and LAB collaborate in anti-fibrotic mechanisms through the induction of PPAR-γ [132,133,140]. Specifically, SCFA-producing bacteria can induce PPAR-γ, while LAB create a microenvironment conducive to the growth of the SCFA-producing bacteria. Currently, there is insufficient strong data to support the routine use of LAB and SCFA-producing bacteria in the therapy of all patients with IBDs. However, one of the most promising therapeutic strategies appears to be modulating these bacteria to intervene in the anti-fibrotic mechanisms controlled by PPAR-γ. Challenges such as the instability of purified microbial metabolites [183], the differing pathophysiology between CD and UC, and the varied impact of fibrosis in these two conditions currently hinder its application.

The lack of data related to the study of metabolites as mediators of intestinal fibrogenesis should be addressed, initially with preclinical studies and subsequently with clinical trials, as they could contribute to the development of potential new therapies complementary to those currently available. The interest addressed to preventive strategies and/or alternative/complementary therapeutic strategies based on prebiotics, probiotics, and postbiotics finds place in the fact that current therapies, although effective, do not lack adverse effects [184]. Further studies on microbial metabolites could not only enhance the understanding of IBD pathogenesis and their treatment but also contribute to a better understanding and treatment of other common and dangerous conditions characterized by fibrosis that can potentially affect any organ.

## 7. Methods

The search reported in Table 4 was performed at the end of March 2024 in the PubMed database and filtered for the last five years, 2020–2024. English-language original papers, short communications, clinical trials, randomized controlled trials, meta-analyses, letters, editorials, and articles were evaluated. Emphasis was placed on the selection of original papers and randomized controlled trials whenever possible. A total of 280 were acquired with the PubMed search; after the duplicates were removed and titles/abstracts were checked, a total of 38 articles were selected for full-text reading. From the reference list of those articles, the authors selected another 27 papers, based on scientific and clinical relevance, resulting in a total number of 65 publications.

## Figures and Tables

**Table 1:** Main changes in composition of microbiota in IBDs.

Phylum	Class	Order	Family	Genus	Species	CD	Ref.	UC	Ref.
*Firmicutes*	*Clostridia*					↓	[9]	↓	[9]
		*Clostridiales*	*Lachnospiraceae*	*Roseburia*	*R. hominis*			↓	[10]
					*R. intestinalis*	↓	[11]	↓	[11]
				*Ruminococcus*	*R. albus*	↓	[12]		
					*R. callidus*	↓	[12]		
					*R. bromii*	↓	[12]		
					*R. gnavus*	↑	[13]	↑	[13]
					*R. torques*	↑	[13]	↑	[13]
			*Acidaminococcaceae*	*Dialister*	*D. invisus*	↓	[14]		
			*Eubacteriaceae*	*Eubacterium*	*E. rectale*	↓	[12]		
			*Clostridiaceae*	*Clostridium*	*C. difficile*	↑	[12]		
					*C. coccoides*	↓	[15]	↓	[15,16]
					*C. leptum*	↓	[12,15,16]	↓	[15]
				*Faecalibacterium*	*F. prausnitzii*	↑	[17]	↓	[10,11,15]
						↓	[11,12,14,15]		
	*Bacilli*					↑	[9]	↑	[9]
		*Bacillales*	*Listeriaceae*	*Listeria*		↑	[12]		
		*Lactobacillales*	*Enterococcaceae*	*Enterococcus*		↑	[12]		
			*Lactobacillaceae*	*Lactobacillus*		↑ ↓	[12,18] [19]	↓	[20]
*Bacteroidetes*	*Bacteroidetes*					↓	[9]	↓	[9]
		*Bacteroidales*	*Bacteroidaceae*	*Bacteroides*	*B. fragilis*	↓	[11,12]	↓	[11]
						↑	[21]		
					*B. vulgatus*	↓	[11,12]	↓	[11]
						↑	[21]		
*Actinobacteria*	*Actinobacteria*					↑	[9]	↓	[22]
		*Bifidobacteriales*	*Bifidobacteriaceae*	*Bifidobacterium*	*B. longum*	↑	[11]		
					*B. bifidum*			↓	[22]
*Proteobacteria*						↑	[9]	↑	[9]
	*δ*	*Desulfovibrionales*	*Desulfovibrionaceae*	*Desulfovibrio*				↑	[23]
	*γ*	*Enterobacteriales*	*Enterobacteriaceae*	*Escherichia*		↑	[11,21]		
				*Shigella*		↑	[11]		
					*S. flexneri*	↑	[12]		
		*Pseudomonadales*	*Moraxellaceae*	*Acinetobacter*	*A. junii*	↑	[21]		
*Verrucomicrobia*	*Verrucomicrobiae*					↓	[13,24]	↓	[13,24]
		*Verrucomicrobiales*	*Verrucomicrobiaceae*	*Akkermansia*	*A. muciniphila*	↓	[13,24]	↓	[13,24]

Legend: ↓ depleted; ↑ enriched. Abbreviations: CD, Crohn’s disease; UC, ulcerative colitis. Background colors denote shifts in phylum/class abundance, with species details listed below. Only relevant species implicated in IBD pathogenesis are featured. Interpretation should acknowledge limitations, including diverse detection methods, samples from varied intestinal sites, and absence of disease activity and therapy specifications. Taxonomy follows traditional nomenclature, as recent changes proposed by the International Committee on Systematics of Prokaryotes (ICSP) [25] remain generally unadopted.

**Table 2:** The main microbial postbiotics, related precursors, microbiota species involved in their metabolism, and their effects on the homeostasis of the four components of the gut barrier in healthy conditions.

			Main Effects on the Parts of the Gut Barrier				
Microbial Metabolites	Precursor	Species Involved in the Metabolism	Microbiota	Mucus	Epithelium	IIS	Ref.
**Short-chain fatty acids (SCFAs)** Acetate Propionate Butyrate	Non-digestible dietary fibers, amino acids, and lactate.	*A. muciniphila* → propionate. *Clostridium* spp., *R. bromii*, *F. prausnitzii*, and *E. rectale* → butyrate.	SCFAs interact with other bacteria such as *Lactobacilli* and *Bifidobacteria*, enhancing their growth.	SCFAs stimulate goblet cells and induce the MUC2 gene.	SCFAs are the principal energetic source for colonocytes and contribute to the integrity of the APC.	SCFAs regulate TLR and FFAR activation, the differentiation of Tregs, and IL-10 secretion.	[70,71,72,73]
**Lactic acid (LA)**	Fermented foods: carbohydrate fermentation.	“LAB”, Gram-positive catalase-negative bacteria resistant to low pH, mainly belonging to the *Lactobacillus* genus.	LAB produce bacteriocins, peptides involved in the mucosal defense.	Various strains of LAB differently affect goblet cell functions and the expression of mucus-related genes, MUC2 included.	LA promotes the TCA for energy production, maintains the cellular redox state, stimulates the ACC for fatty acid synthesis, and contributes to normal epithelial proliferation.	LAB administration promotes macrophage M2 polarization and a reduction in pro-inflammatory cytokines (e.g., IL-1β and IL-6)	[74,75,76,77]
**Indoles**	Tryptophan, the essential amino acid found in meat, fish, dairy, eggs, nuts, seeds, legumes, and whole grains.	Tryptophanase-expressing bacteria, such as *Clostridium*, *Bacteroides*, *Lactobacillus*, and *Bifidobacterium* spp.	Indoles influence bacterial communication, limiting virulence gene expression and bacterial invasiveness, in a dose-dependent manner.	Indoles boost MUC2 and MUC4 expression and goblet cell activity.	Indoles reduce the epithelial permeability by enhancing tight junctions.		[78,79,80,81,82]
**Urolithin A (UA)**	Polyphenolic compounds (ellagitannins) in fruits, nuts, and tea.	In the small intestine, ellagitannins are hydrolyzed to ellagic and gallic acid intermediates, and further metabolized by *Gordonibacter urolithinfaciens* and *Ellagibactrer* into UA. Only about 40% of elderly humans possess a suitable gut microbiota able to produce UA.			UA and its synthetic analogue, UAS03, have been reported to upregulate tight junction proteins.	UA reduces the production of ROS and suppresses the TLR4, MAPK, and PI3K pathways, with decrease in the expression of pro-inflammatory mi-RNA and cytokines (IL-1β, IL-6, and TNF-α).	[83,84,85]
**Hydrogen sulfide (H_2_S)**	Sulfate (SO_4_^2−^) derived from amino acids (mainly cysteine and methionine), additives, preservatives, and IEC production (CBS activity).	Sulfate-reducing bacteria (SRB), like colonic *Desulfovibrio*, *Desulfotomaculum*, and *Bilophila*, utilize SO_4_^2−^ as a terminal electron acceptor in their metabolic pathways, reducing it to H_2_S.	Exogenous H_2_S confers to the bacteria’s high resistance to oxidative stress.	High concentrations of H_2_S destabilize the disulfide bonds of the mucin-2 network, resulting in increased contact between bacteria and the epithelium.	H_2_S is the primary mineral energy substrate for colonocytes, but in high concentrations, it inhibits the mitochondrial respiratory chain. Also, it negatively interferes with butyrate metabolism.		[86,87,88,89,90,91,92]
**Trimethylamine (TMA)**	Choline, carnitine, and betaine, contained in red meat, eggs, fish, and dairy.	Several bacterial species (e.g., *E. coli*, *Enterococcus*, *Clostridium*, *Proteus*, *Shigella*, *Klebsiella*, and *Providentia* spp.) transform the precursors in TMA, which is further oxidized in the liver to form TMAO.	TMA and TMAO modulate the composition of the microbiota.				[93,94]

Abbreviations: ACC, acetyl-CoA carboxylase; FFARs, free fatty acid receptors; H_2_S, hydrogen sulfide; IECs, intestinal epithelial cells; IIS, intestinal immune system; LAB, lactic acid bacteria; LA, lactic acid; SCFA, short-chain fatty acid; SRB, sulfate-reducing bacteria; TCA, tricarboxylic acid cycle; TLRs, Toll-Like Receptors; TMA, trimethylamine; TMAO, trimethylamine-N-oxide; Trp, tryptophan; UA, urolithin A.

## Data Availability

Data sharing is not applicable.
